# Sex differences in the association between socioeconomic status and untreated hypertension among residents with hypertension in rural Khánh Hòa, Vietnam: a post-hoc analysis

**DOI:** 10.1186/s12872-024-03706-4

**Published:** 2024-01-20

**Authors:** Yuta Yokobori, Ami Fukunaga, Sumiyo Okawa, Masahiko Hachiya, Chau Que Nguyen, Thuy Phuong Thi Pham, Dong Van Hoang, Danh Cong Phan, Dong Van Huynh, Huy Xuan Le, Hung Thai Do, Tetsuya Mizoue, Yosuke Inoue

**Affiliations:** 1 Bureau of International Health Cooperation, National Center for Global Health and Medicine, 1-21-1, Toyama, Shinjuku-ku, Tokyo, Japan; 2 Department of Epidemiology and Prevention, Center for Clinical Sciences, National Center for Global Health and Medicine, Tokyo, Japan; 3 Department of Non-communicable Disease Control and Nutrition, Pasteur Institute in Nha Trang, Nha Trang, Khánh Hòa Vietnam; 4 Khánh Hòa Center for Disease Control, Nha Trang, Khánh Hòa Vietnam; 5 Pasteur Institute in Nha Trang, Nha Trang, Khánh Hòa Vietnam

**Keywords:** Untreated hypertension, Socioeconomic status, Income, Education, Vietnam

## Abstract

**Background:**

Several studies have examined the association between socioeconomic status (SES) and the proportion of untreated hypertension, but have produced conflicting findings. In addition, no study has been conducted to determine sex differences in the association between SES and untreated hypertension. Thus, the aim of this study was to examine whether the associations between SES and the proportion of untreated hypertension differed by sex in Vietnam.

**Methods:**

This study was conducted using the data of 1189 individuals (558 males and 631 females) who were judged to have hypertension during the baseline survey of a prospective cohort study of 3000 residents aged 40–60 years in the Khánh Hòa Province. A multilevel Poisson regression model with a robust variance estimator was used to examine whether sex and SES indicators (household income and educational attainment) interacted in relation to untreated hypertension.

**Results:**

The proportion of untreated hypertension among individuals identified as hypertensive was 69.1%. We found significant interaction between sex and SES indicators in relation to untreated hypertension (education: *p* < 0.001; household income: *p* < 0.001). Specifically, the association between SES and untreated hypertension was inverse among males while it was rather positive among females.

**Conclusions:**

Our finding suggests that the role of SES in the proportion of untreated hypertension might differ by sex.

**Supplementary Information:**

The online version contains supplementary material available at 10.1186/s12872-024-03706-4.

## Background

Although hypertension can easily be detected through the measurement of blood pressure and can often be treated effectively using medications, 700 million cases are estimated to be untreated globally [1, 2]. As 62% of all cases of cerebrovascular diseases and 49% of ischemic heart diseases are estimated to be attributable to suboptimal control of blood pressure [3, 4], untreated hypertension has long been a topic of interest among researchers and practitioners [5–7]. The proportion of individuals who take antihypertensive treatment or counselling among those at a higher risk of cardiovascular disease has been employed by the NCD Global Monitoring Framework coordinated by the World Health Organization when they monitor progress to reduce disease burden associated with NCD in each country [8].

Given that socioeconomic status (SES), e.g., income and education, has been widely recognized as the fundamental causes of a wide range of health outcomes, several studies have examined the association between SES and untreated hypertension, but have produced conflicting findings [9]. Previous studies conducted in the Netherlands [10], the United Kingdom [11], Switzerland [12], and Korea [13] reported a positive association between SES and untreated hypertension. For example, Petersen et al. examined the association among 8933 participants in the UK and suggested that frequent hospital visit among those who were impoverished might have underlain this social gradient in untreated hypertension [11]. On the other hand, inverse associations were reported in Singapore [14], Iran [15], Bangladesh [16], Russia [17], India [18], and Tanzania [19], which was explained by a better access to healthcare services among those with high SES. There were also several studies that reported the null findings [20–24]. It is possible that different indicators used in each study might have underlain the reported inconsistency. In addition, the aspects of SES captured by these indicators and the way such aspects affect health can be different across study populations (which can differ in terms of age, sex, country, and time).

This study was designed to extend the previous studies by examining the association between SES and untreated hypertension in rural Vietnam while focusing on sex differences in the association. There are several reasons we believe this study is important. First, sex is a factor that could modify the association between SES and several health outcomes [25, 26], as exemplified by Wu et al. that reported the association between educational attainment and diabetes to be positive and inverse among male and female participants, respectively [27]. However, little attempt has been made to examine whether the association between SES and untreated hypertension differs according to sex. Second, more research should be conducted in low- and middle-income countries (LMICs) on the association between SES and untreated hypertension. In a previous study conducted in 17 LMICs, only a quarter of the patients with known hypertension (25%) reported receiving pharmaceutical drugs [28, 29]. Clarifying the correlation between SES and untreated hypertension in LMICs is of utmost importance because early detection and prevention of cardiovascular disease (CVD) is the key to reducing disease burden in LMICs. In doing so, we also examined whether the associations between different SES indicators, such as income and education, and untreated hypertension varied. It is possible that the effects of high SES on health/health-related behaviors are different or even detrimental depending on how individuals achieve their socioeconomic success, particularly in LMICs [30].

Therefore, the aim of this study was to examine the sex-specific associations between SES and untreated hypertension in rural Vietnam, with special focus on variations in the association between the indicators of SES (i.e., income and educational attainment) and the proportion of untreated hypertension.

## Methods

### Study setting

The data used for this study were obtained from the baseline survey of the Khánh Hòa Cardiovascular Study (KHCS), which was conducted between June 2019 and June 2020. The aim of the KHCS was to examine the determinants of CVD risk factors among the middle-aged population in rural communes of Vietnam. Eight communes were selected from one district of the Khánh Hòa Province. Thereafter, the staff of the health center in each commune created a list of residents aged 40–59 years at the time of recruitment and invited them to participate in the study (participation rate: 75–87%). The exclusion criteria were as follows: (1) those who have lived in the commune for less than 6 months; (2) institutionalized people; (3) those who were unable to provide informed consent; (4) those who planned to move out of the community within 1 year; (5) pregnant women and those who gave birth within a year; and (5) those with a history of CVD events.

For this study, we confined our analytic sample to those who were judged to have hypertension. Of the 3000 participants included in the KHCS, we included those who had a systolic blood pressure of ≥140 mmHg, diastolic blood pressure of ≥90 mmHg or were taking any antihypertensive medication [31], which resulted in an analytic sample of 1189 participants (558 males and 631 females).

### Outcomes

Blood pressure was measured twice using an electric sphygmomanometer (Omron, HEM1020, Tokyo, Japan). The measurements were taken with the participants seated and their arms supported at the heart level. The participants were instructed to rest for at least 5 min prior to the first measurement. The two measurements were used to calculate mean systolic and diastolic blood pressure.

Hypertension was defined as a systolic blood pressure of ≥140 mmHg, diastolic blood pressure of ≥90 mmHg, or self-reported use of antihypertensive medication. Participants with hypertension who did not self-report the use of antihypertensive medication within the last 2 weeks were defined as those with untreated hypertension (irrespective of diagnostic and screening status).

### Exposure variables

Educational attainment and household income were employed as SES indicators. Regarding educational attainment, participants were asked to report their highest educational attainment using the following response options: no school, did not complete primary school, completed primary school, secondary school, senior high school, college/university, and postgraduate. The responses were then categorized into three levels: primary school and below, secondary school, and high school and above.

Participants reported their estimated monthly household income by choosing one response options out of 10 that best described their income (ranging from ≤1,000,000 VND to > 20,000,000 VND and do not know). We calculated the equivalized income by using the information on household income and the number of household members, which was then categorized into low (< 3,130,495 VND, equivalent to < 136.1 USD), middle (3162278–5,000,000 VND, equivalent to 137.5–217.4 USD), and high (≥5,291,503 VND, equivalent to ≥230.0 USD)) at the exchange rate in January 2019 [32].

### Covariates

We extracted information on sociodemographic variables, including age (40–44 years, 45–49 years, 50–54 years, and 55–60 years), sex (man or woman), job categories (government employee, non-governmental employee, self-employed, farmer or fisherman, homemaker, other or unemployed), and marital status (married/cohabiting or not married), from questionnaire responses. A participant was diagnosed with diabetes mellitus (DM) per the following American Diabetes Association criteria of 2019: fasting plasma glucose ≥7 mmol/l (≥ 126 mg/dL), HbA1c ≥ 6·5% or self-reported use of antidiabetic medication. Dyslipidemia was defined as total cholesterol ≥6.22 mmol/l or LDL-cholesterol ≥4.14 mmol/l or HDL-cholesterol < 1.03 mmol/l or Triglyceride ≥2.26 mmol/l or self-reported use of antihyperlipidemic medication. In addition, the 11-item Center for Epidemiologic Studies Depression Scale was used to define depressive symptoms, with a score of ≥9 indicating depressive symptoms [33–35].

### Statistical analysis

We performed multiple imputation to handle missing data on household income (*n* = 33) based on the age, sex, marital status, and education level of each of the 3000 participants of the KHCS. The pooled data of 20 imputation sets were used for the analysis. We used the chained equation method with 200 iterations, and a linear regression model was used to impute the data. The imputation estimates were combined using Rubin’s rules [36].

A multilevel Poisson regression model with robust variance estimator was used to examine if SES indicators (i.e., educational attainment and household income) and sex interact in relation to untreated hypertension, while accounting for possible heterogeneity at the community level (Level 1: individual, Level 2: community). When one SES indicator and its interaction term with sex were incorporated in the model, we also adjusted for the other SES indicator in the model. The models were adjusted for age, marital status, occupation, DM, dyslipidemia, and depressive symptoms.

We also conducted a set of sensitivity analysis in which we confined our analytic sample to those who were diagnosed with hypertension by doctors (*n* = 527). Models were adjusted in the same manner as in the main analysis described above.

Results are presented as prevalence ratios (PRs) and corresponding 95% confidence intervals (CIs) of untreated hypertension. All statistical analyses were conducted using Stata ver. 16.0 (College Station, TX, USA).

## Results

The basic characteristics of the study participants are outlined in Table 1. Of the 1189 participants, 53.1% were female. The proportion of females with a low educational attainment (62.4%) was higher than that of females with a high educational attainment (37.8%). The proportions of the female participants in each income group were comparable. The age distribution and marital statuses of the participants at each SES level were analogous. The proportion of the total population with depressive symptoms was 9.3%, with lower proportions observed at the higher SES level than at the *lower* SES *level.*

**Table 1:** Basic characteristics of individuals with hypertension who participated in the baseline survey of the Khánh Hòa Cardiovascular Study (2019–2020) (*n* = 1189)

	All participants (*n* = 1189)	Educational attainment			Household income^a^		
		Low (*n* = 505)	Middle (*n* = 409)	High (*n* = 275)	Low (*n* = 410)	Middle (*n* = 411)	High (*n* = 356)
Sex (female), n (%)	631 (53.1)	315 (62.4)	212 (51.8)	104 (37.8)	240 (58.5)	208 (50.6)	175 (49.2)
Age (years), n (%)							
40–44	148 (12.5)	64 (12.7)	53 (13.0)	31 (11.3)	46 (11.2)	56 (13.6)	44 (12.4)
45–49	276 (23.2)	108 (21.4)	108 (26.4)	60 (21.8)	97 (23.7)	99 (24.1)	78 (21.9)
50–54	369 (31.0)	148 (29.3)	127 (31.1)	94 (34.2)	129 (31.5)	126 (30.7)	111 (31.2)
55–60	396 (33.3)	185 (36.6)	121 (29.6)	90 (32.7)	138 (33.7)	130 (31.6)	123 (34.6)
Job, n (%)							
Government Employee	295 (9.8)	6 (0.5)	30 (2.8)	259 (36.1)	18 (1.8)	101 (9.7)	176 (19.1)
Non-Government Employee	483 (16.1)	189 (15.6)	200 (18.7)	94 (13.1)	164 (16.4)	191 (18.3)	124 (13.5)
Informal Sector	2103 (70.1)	996 (82.0)	812 (76.0)	295 (41.1)	794 (79.2)	723 (69.2)	557 (60.5)
Unemployed	119 (4.0)	24 (2.0)	26 (2.4)	69 (9.6)	26 (2.6)	30 (2.9)	63 (6.9)
Marital status (married/cohabiting), n (%)	1058 (89.0)	434 (85.9)	373 (91.2)	251 (91.3)	352 (85.9)	372 (90.5)	325 (91.3)
Diabetes mellitus, n (%)	177 (14.9)	81 (16.0)	57 (13.9)	39 (14.2)	71 (17.3)	50 (12.2)	56 (15.7)
Dyslipidemia, n (%)	655 (55.1)	279 (55.3)	216 (52.8)	160 (58.2)	210 (51.2)	229 (55.7)	210 (59.0)
Depressive symptoms, n (%)	110 (9.3)	64 (12.7)	35 (8.6)	11 (4.0)	56 (13.7)	33 (8.0)	19 (5.3)

^a^Twelve individuals had missing income data

**Fig. 1:**
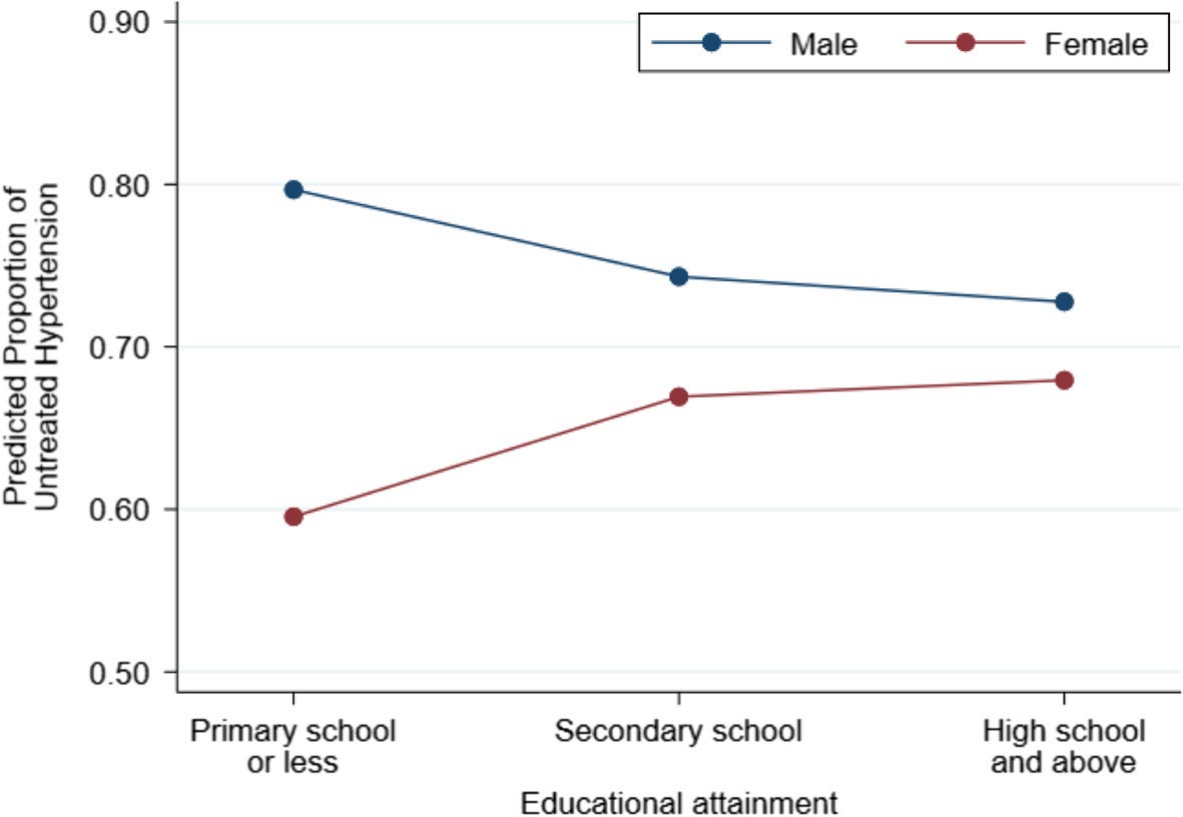
Predicted proportions of untreated hypertension at given combinations of sex and educational attainment

**Fig. 2:**
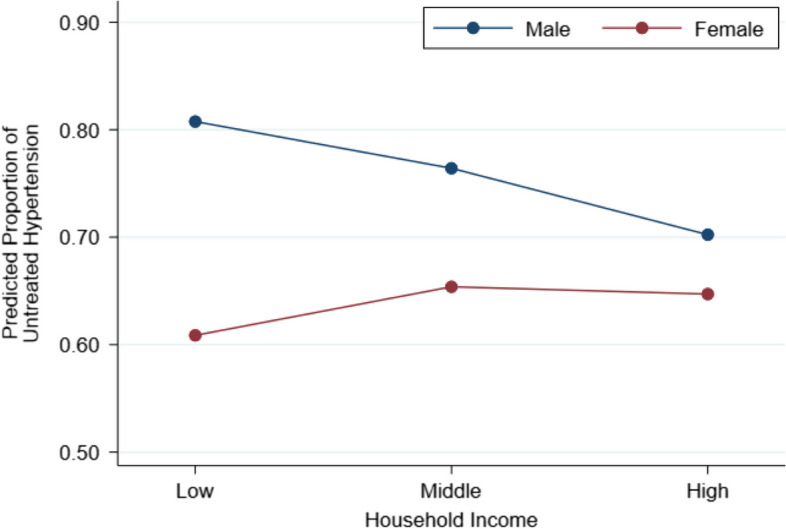
Predicted proportions of untreated hypertension at given combinations of sex and household income

Of the individuals included in this study, 822 (69.1%, 95%CI = 66.4–71.6) did not use antihypertensive medications (i.e., untreated hypertension). We also show that the proportion of untreated hypertension differed by sex and SES indicator categories in Supplementary Table 1. Table 2 shows the results of a multilevel Poisson regression analysis with a robust variance estimator of the association between SES indicators and the proportion of untreated hypertension. The significant interaction terms between sex and SES indicators indicated that the association between SES and the proportion of untreated hypertension differed according to sex (*p* < 0.001 for the interaction in relation to educational attainment and household income). As illustrated in Fig. 1, educational attainment was positively associated with the proportion of untreated hypertension among the female participants (PR = 1.12, 95%CI = 1.04–1.22 for those who completed secondary school vs. those who completed primary school or below; PR = 1.11, 95%CI = 0.97–1.28 for those who graduated from high school or attained further education). On the other hand, among male participants, the SES indicator was inversely associated with the proportion of untreated hypertension. When the males in the lowest educational attainment group were set as the reference, the PRs were 0.93 (95%CI = 0.89–0.97) and 0.93 (95%CI = 0.84–1.03) for those in the middle and highest educational attainment groups, respectively (data not shown in Table). When SES was defined based on household income, we also observed an inverse association among males and positive association between females while the latter was not statistically significant (Model 2 and Fig. 2).

**Table 2:** Results of Poisson regression models with a robust variance estimator examining the interaction between sex and socioeconomic status indicators in relation to untreated hypertension among individuals with hypertension in rural Khanh Hoa, Vietnam (2019–2020)

	The number of individuals with untreated hypertension, n (%)	Model 1	Model 2
Sex			
Male	421 (75.5%)	1.00 (Ref.)	1.00 (Ref.)
Female	491 (63.6%)	0.74 (0.69–0.80)	0.76 (0.68–0.84)
Education			
Primary school	339 (67.1%)	1.00 (Ref.)	1.00 (Ref.)
Secondary school	290 (70.9%)	0.93 (0.89–0.97)	1.04 (0.97**–**1.10)
High school or higher	193 (70.2%)	0.92 (0.83–1.02)	1.02 (0.93**–**1.11)
Household income			
Low	280 (68.3%)	1.00 (Ref.)	1.00 (Ref.)
Middle	294 (71.5%)	1.01 (0.95**–**1.08)	0.95 (0.87**–**1.04)
High	240 (67.4%)	0.96 (0.86**–**1.08)	0.87 (0.76–0.98)
Sex × Education			
Female × Primary school		1.00 (Ref.)	
Female × Secondary school		1.21 (1.14–1.28)	
Female × High school or higher		1.24 (1.05–1.47)	
Sex × Household income			
Female × Low			1.00 (Ref.)
Female × Middle			1.13 (0.97**–**1.31)
Female × High			1.22 (1.12–1.33)

Data are expressed as prevalence ratio (95% confidence interval)

Models were adjusted for age, marital status, occupation, DM, dyslipidemia, and depressive symptoms

Community was included as a cluster in all models

When we confined our study participants to those who had been previously diagnosed hypertension, we found inverse associations between SES and untreated hypertension among males (only when we used household income to define SES) and positive associations among females (Supplementary Table 2 and Supplementary Figs. 1 and 2). The inverse association observed in the main analysis between educational attainment and untreated hypertension among males was not observed in the sensitivity analysis (Supplementary Fig. 1).

## Discussion

In this study of 1189 middle-aged community dwellers with hypertension in rural Khánh Hòa, Vietnam, we found that the association between SES and the proportion of untreated hypertension differed according to sex. Although we did not find any strong evidence of a significant association between SES and the proportion of untreated hypertension among the female participants, the male participants with lower vs. higher SES were more likely to have untreated hypertension. These associations did not materially change when they were analyzed using the two SES indicators (educational attainment and household income).

The inverse association between SES and the proportion of untreated hypertension among the male participants in the present study is in line with the findings reported in previous systematic reviews on the association between SES and the proportion of untreated hypertension in LMICs [37]. Gupta et al. [38] reported that among 33,423 participants from 150 communities in India, Pakistan, and Bangladesh, greater household wealth and higher education were inversely associated with the proportion of untreated hypertension although they did not examine sex-specific associations in their study. A possible interpretation for this inverse association is that health literacy and knowledge of medications may be better among those with higher educational attainment than among those with lower educational attainment. Furthermore, it is possible that commune members expect individuals with higher SES to be good role models and adhere to appropriate medication use. This supposition is supported by the findings of previous studies conducted in Asia [39, 40], which indicated that individuals in a socially important position are more likely to feel responsible for adherence to social norms.

In our study, higher SES was associated with lower proportion of untreated hypertension among the male participants, but not among the female participants. A possible interpretation for this discrepancy is that females in rural Vietnam have access to healthcare services or health information irrespective of their SES. In the health system of Vietnam, village health workers, who are usually females, play critical roles in promoting primary healthcare at the commune level. They often organize health education campaigns and mobilize residents (particularly female residents) to discuss health issues. Under such circumstances, it is possible that health-related information is diffused faster and more efficiently among females across different SES groups than among males. This is in line with the findings of a previous study conducted in Asia, which demonstrated that females with dense networks are likely to exhibit an elevated level of hypertension awareness and control; however, this finding was not observed in males [40].

Based on the information in the review by Howe et al., we hypothesized that education reflects the level of cognitive ability to access health education messages or appropriate health services, leading to hypertension treatment, whereas income, which is an indicator of material resources, is not necessarily linked with hypertension treatment [30, 41]. In this study, educational attainment and household income may have captured the SES gradient in a similar manner (Spearman’s rho = 0.2761; *p* < 0.001). Although our original hypothesis was that the correlation between educational attainment or household income and untreated hypertension vary, the results of the present study indicated that the associations between them were not significantly different.

Of the total study participants in the KHCS (mean age: 49.9 years old), those with hypertension occupied nearly 40% [42], which is comparable to or slightly higher than the Vietnamese population average estimated by the NCD RisC; the hypertension prevalence for 45–49 and 50–54 years old was estimated to be 31.1 and 37.9% for males and 23.0 and 31.3% for females, respectively [43]. Although economic development/urbanization in the past few decades observed in LMICs seemingly have contributed to the increasing prevalence of individuals with hypertension, people in such countries still have limited access to medical/public health care services or health education. The small proportion of treated individuals observed in this study (30.9%) can be regarded as one such example. When policy makers develop action plans to expand medication coverage, the role of SES on hypertension treatment and a possible difference in the association between males and females should be carefully considered.

This study has several limitations. First, the information on hypertension treatment was self-reported; thus, it may be subject to social desirability bias or recall bias. Second, we only focused on pharmaceutical treatment and did not collect information on whether some participants were instructed to modify their diets or physical activities. The observed association between SES and untreated hypertension may have been different if we accounted for such individuals. Third, we only used two SES indicators (i.e., educational attainment and household income) while SES is a multifaceted construct that can be explained by several other aspects (e.g., wealth, and social prestige). Lastly, our study population may not fully represent the individuals with hypertension in Vietnam. Thus, caution should be exercised when generalizing the results of this study to other populations.

## Conclusion

This study demonstrated that SES is inversely associated with untreated hypertension among males. In addition, the results of this study showed that the proportion of untreated hypertension among females is generally lower than that among males, regardless of their SES. These findings suggest the importance of identifying those at higher risk of untreated hypertension when formulating policies for hypertension management in rural areas of Vietnam.

### Supplementary Information

**Additional file 1: Supplementary Table 1.** Prevalence of untreated hypertension among individuals with hypertension in the Khanh Hoa Cardiovascular Study (2019-2020), shown by sex and socioeconomic status categories.

**Additional file 2: Supplementary Table 2.** Results of Poisson regression models with a robust variance estimator examining the interaction between sex and socioeconomic status indicators in relation to untreated hypertension among individuals diagnosed with hypertension by doctors in rural Khanh Hoa, Vietnam (2019–2020).

**Additional file 3: Supplementary Figure 1.** Predicted proportions of untreated hypertension according to sex and educational attainment among people diagnosed with hypertension by doctors.

**Additional file 4:Supplementary Figure 2.** Predicted proportions of untreated hypertension according to sex and income level among people diagnosed with hypertension as doctors.

## Data Availability

The data are not publicly available but are available upon reasonable request to the corresponding author.

## Abbreviations

CI: Confidence interval
CVD: Cardiovascular disease
DM: Diabetes mellitus
KHCS: Khánh Hòa Cardiovascular Study
LMIC: Low- and middle-income country
NCD: Non-communicable diseases
NCGM: National Center for Global Health and Medicine
PR: Prevalence ratio
SES: Socioeconomic status
