# Supplementary material for: Sex differences in the association between socioeconomic status and untreated hypertension among residents with hypertension in rural Khánh Hòa, Vietnam: a post-hoc analysis
Source: BMC Cardiovasc Disord. 2024 Jan 20;24:61. doi: 10.1186/s12872-024-03706-4 (PMC10799502; doi:10.1186/s12872-024-03706-4)
Supplement: Supplementary file 1 — Additional file 1: Supplementary Table 1. Prevalence of untreated hypertension among individuals with hypertension in the Khanh Hoa Cardiovascular Study (2019-2020), shown by sex and socioeconomic status categories. [file 12872_2024_3706_MOESM1_ESM.docx]

**Supplementary Table 1.** Proportions of untreated hypertension among individuals with hypertension in the Khanh Hoa Cardiovascular Study (2019-2020), shown by sex and socioeconomic status categories.

|  | All  n/total (%) | Male  n/total (%) | Female  n/total (%) |
| --- | --- | --- | --- |
| Total | 822/1189 (69.1) | 421/558 (75.5) | 401/631 (63.6) |
| Education |  |  |  |
| Primary school and below | 339/505 (67.1) | 153/190 (80.5) | 186/315 (59.1) |
| Secondary school | 290/409 (70.9) | 145/197 (73.6) | 145/212 (68.4) |
| High school or higher | 193/275 (70.2) | 123/171 (71.9) | 70/104 (67.3) |
| Household income^1^ |  |  |  |
| Low | 280/410 (68.3) | 134/170 (78.8) | 146/240 (60.8) |
| Middle | 294/411 (71.5) | 158/203 (77.8) | 136/208 (65.4) |
| High | 240/356 (67.4) | 127/181 (70.2) | 113/175 (64.6) |

^1^ Information was available for 1,177 individuals, with missing data for 6 male and 27 female participants.
