# Supplementary material for: Sex differences in the association between socioeconomic status and untreated hypertension among residents with hypertension in rural Khánh Hòa, Vietnam: a post-hoc analysis
Source: BMC Cardiovasc Disord. 2024 Jan 20;24:61. doi: 10.1186/s12872-024-03706-4 (PMC10799502; doi:10.1186/s12872-024-03706-4)
Supplement: Supplementary file 2 — Additional file 2: Supplementary Table 2. Results of Poisson regression models with a robust variance estimator examining the interaction between sex and socioeconomic status indicators in relation to untreated hypertension among individuals diagnosed with hypertension by doctors in rural Khanh Hoa, Vietnam (2019–2020). [file 12872_2024_3706_MOESM2_ESM.docx]

**Supplementary Table 2.** Results of Poisson regression models with a robust variance estimator examining the interaction between sex and socioeconomic status indicators in relation to untreated hypertension among individuals diagnosed with hypertension by doctors in rural Khanh Hoa, Vietnam (2019–2020).

|  | Model 1 | Model 2 |
| --- | --- | --- |
| Sex |  |  |
| Male | 1.00 (reference) | 1.00 (reference) |
| Female | 0.64 (0.47–0.89) | 0.46 (0.29- 0.73) |
| Education |  |  |
| Primary school and below | 1.00 (reference) | 1.00 (reference) |
| Secondary school | 0.91 (0.69–1.20) | 0.98 (0.88-1.08) |
| High school or higher | 0.99 (0.60–1.63) | 1.12 (0.72-1.75) |
| Household income |  |  |
| Low | 1.00 (reference) | 1.00 (reference) |
| Middle | 1.16 (0.82-1.66) | 0.79 (0.48–1.30) |
| High | 0.99 (0.63-1.55) | 0.76 (0.39–1.48) |
| Sex × Education |  |  |
| Female × Primary school | 1.00 (reference) |  |
| Female × Secondary school | 1.11 (0.71–1.73) |  |
| Female × High school or higher | 1.32 (0.84–2.06) |  |
| Sex × Household income |  |  |
| Female × Low |  | 1.00 (reference) |
| Female × Middle |  | 2.08 (1.24–3.46) |
| Female × High |  | 1.71 (0.91–3.19) |

Data are expressed as prevalence ratio (95% confidence interval).

Models were adjusted for age, marital status, occupation, diabetes mellitus, dyslipidemia, and depressive symptoms. Community was included as a cluster in all models.
