# Supplementary material for: Sex differences in the association between socioeconomic status and untreated hypertension among residents with hypertension in rural Khánh Hòa, Vietnam: a post-hoc analysis
Source: BMC Cardiovasc Disord. 2024 Jan 20;24:61. doi: 10.1186/s12872-024-03706-4 (PMC10799502; doi:10.1186/s12872-024-03706-4)

**Supplementary Figure 1.** Predicted proportions of untreated hypertension according to sex and educational attainment among people diagnosed with hypertension by doctors.


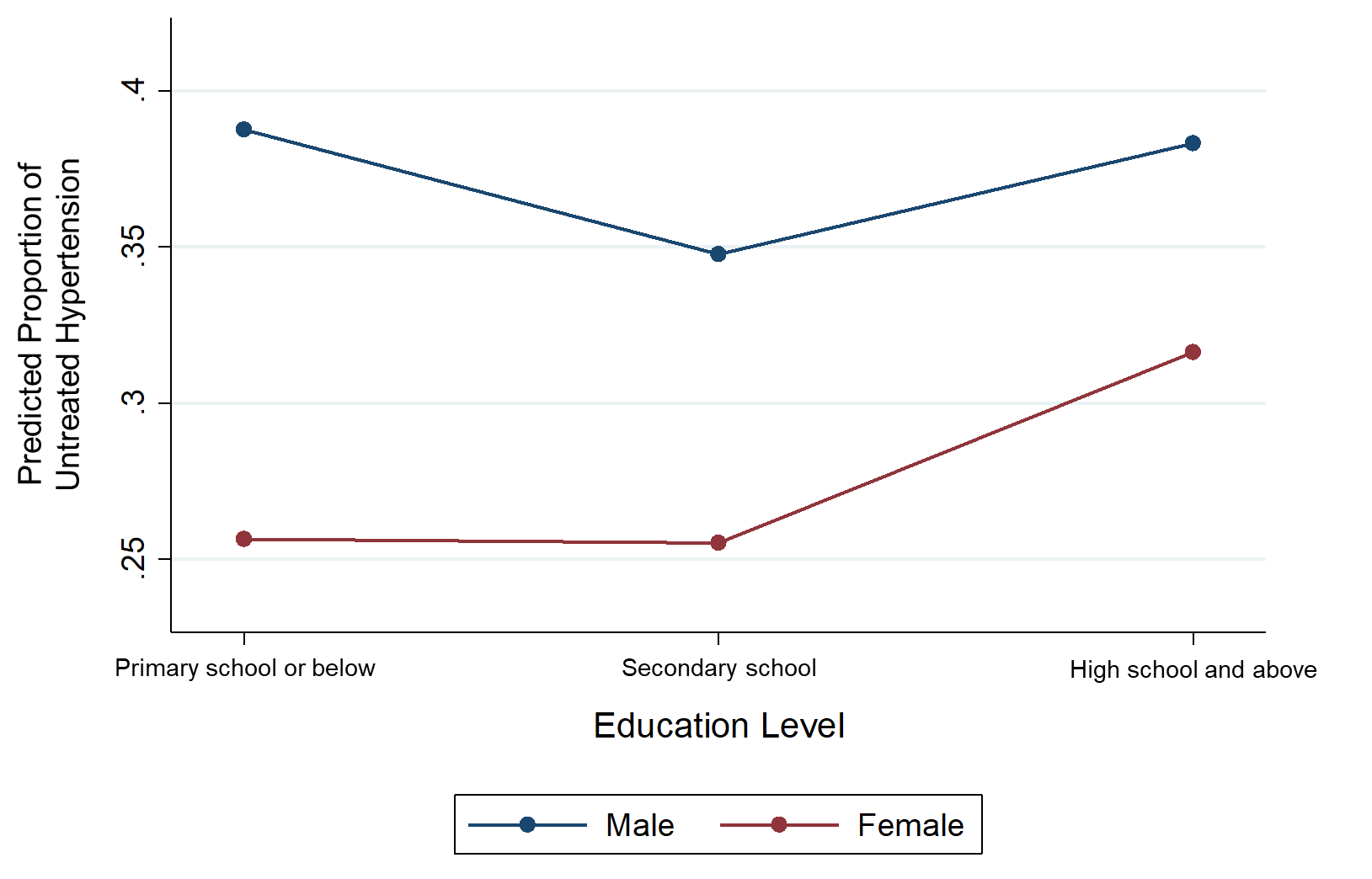

Supplement: Supplementary file 3 — Additional file 3: Supplementary Figure 1. Predicted proportions of untreated hypertension according to sex and educational attainment among people diagnosed with hypertension by doctors. [file 12872_2024_3706_MOESM3_ESM.docx]
