# Supplementary material for: Sex differences in the association between socioeconomic status and untreated hypertension among residents with hypertension in rural Khánh Hòa, Vietnam: a post-hoc analysis
Source: BMC Cardiovasc Disord. 2024 Jan 20;24:61. doi: 10.1186/s12872-024-03706-4 (PMC10799502; doi:10.1186/s12872-024-03706-4)

**Supplementary Figure 2.** Predicted proportion of untreated hypertension according to sex and income level among people diagnosed with hypertension by doctors.


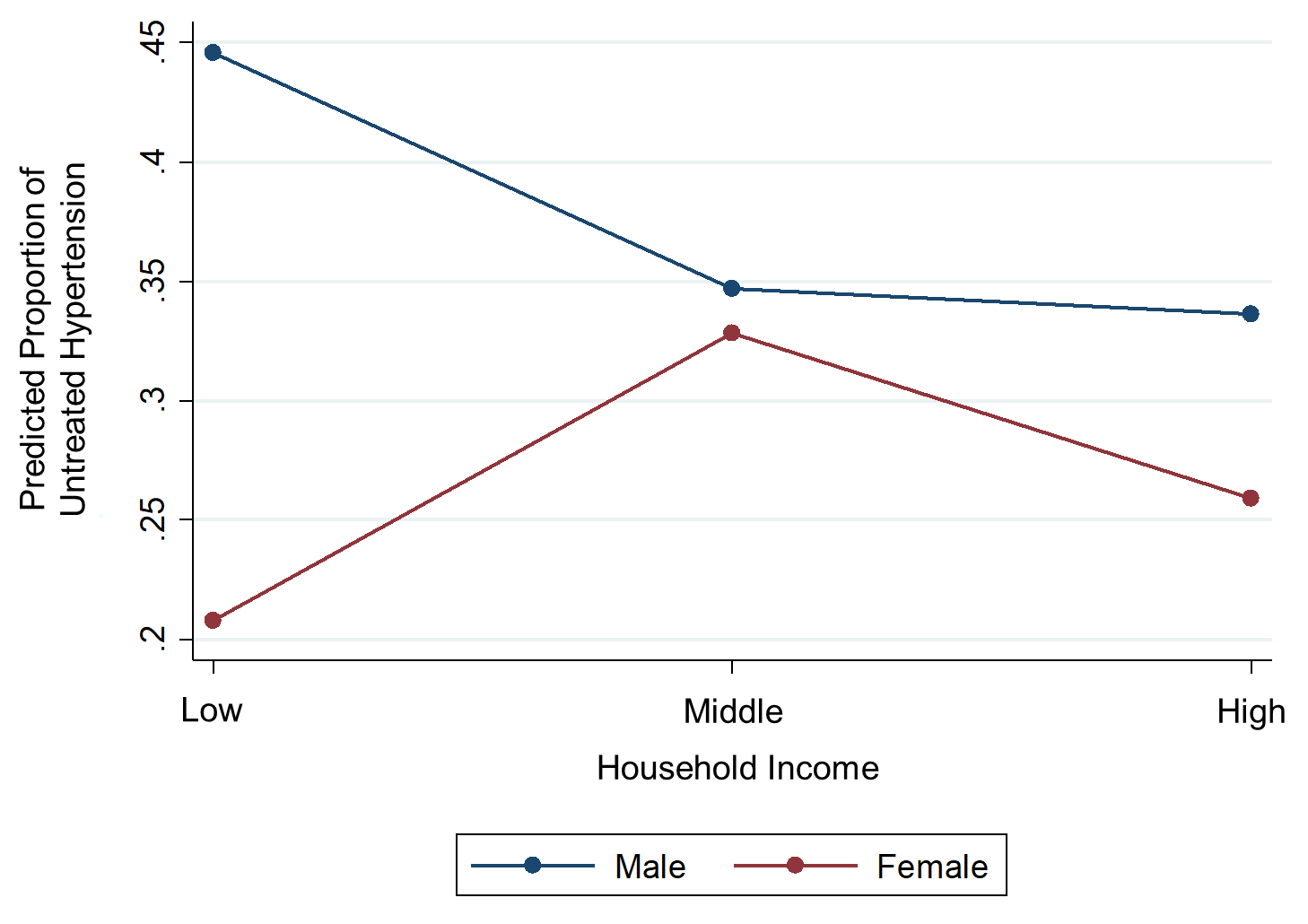

Supplement: Supplementary file 4 — Additional file 4:Supplementary Figure 2. Predicted proportions of untreated hypertension according to sex and income level among people diagnosed with hypertension as doctors. [file 12872_2024_3706_MOESM4_ESM.docx]
